# The threat of antimicrobial resistance in developing countries: causes and control strategies

**DOI:** 10.1186/s13756-017-0208-x

**Published:** 2017-05-15

**Authors:** James A. Ayukekbong, Michel Ntemgwa, Andrew N. Atabe

**Affiliations:** 1 Section for Clinical Microbiology, Redeem Biomedical, P.O. Box 16, Buea, Cameroon; 2 Human Safety Division, Veterinary Drugs Directorate, Health Products and Food Branch, Health Canada, Ottawa, ON Canada; 3 School of Epidemiology, Public Health and Preventive Medicine, Faculty of Medicine, University of Ottawa, Ottawa, Canada; 4 Metabiota Inc., Nanaimo, BC Canada

**Keywords:** Antimicrobial therapy, Developing countries, Microorganisms, Resistance

## Abstract

The causes of antimicrobial resistance (AMR) in developing countries are complex and may be rooted in practices of health care professionals and patients’ behavior towards the use of antimicrobials as well as supply chains of antimicrobials in the population. Some of these factors may include inappropriate prescription practices, inadequate patient education, limited diagnostic facilities, unauthorized sale of antimicrobials, lack of appropriate functioning drug regulatory mechanisms, and non-human use of antimicrobials such as in animal production. Considering that these factors in developing countries may vary from those in developed countries, intervention efforts in developing countries need to address the context and focus on the root causes specific to this part of the world. Here, we describe these health-seeking behaviors that lead to the threat of AMR and healthcare practices that drive the development of AMR in developing countries and we discuss alternatives for disease prevention as well as other treatment options worth exploring.

## Background

The threat of antimicrobial resistance (AMR) is growing at an alarming rate and the situation is perhaps aggravated in developing countries due to gross abuse in the use of antimicrobials [1]. It is well known that any use of antimicrobials however appropriate and justified, contributes to the development of resistance, but widespread unnecessary and excessive use makes the situation worse [2]. Misuse of antimicrobials is facilitated in developing countries by their availability over the counter, without prescription and through unregulated supply chains [1, 3]. Non-compliance in the use of antimicrobials has many repercussions upon resistance and poverty is a major root factor of antimicrobial misuse in developing countries [4]. On the other hand, even among the rich, some patients miss doses either by mistake or deliberate, especially in cases where signs and symptoms begin to subside after an initial favorable therapeutic response [5]. In other situations, such as in the event of an acute side effect, patients abandon their treatment, only to return to the hospital with a recurring infection by a more virulent and resistant strain of the microbe [6]. These actions result in the exposure of surviving pathogens to sub-therapeutic concentrations of antimicrobials thus increasing the chances of acquiring resistance. Self-medication is a common practice in developing countries where patients often get antimicrobials without prescription and through unregulated supply chains [7]. To make the situation even worst, some patients seek their first-line of treatment from traditional healers who provide them with herbal combinations for the treatment of infections. These substances of unknown composition and potency may enhance pathogen fitness and contribute to the development of resistance. Antimicrobial resistance often occur through the inhibition of specific antimicrobial pathways such as cell wall synthesis, nucleic acid synthesis, ribosome function, protein synthesis, folate metabolism, and cell membrane function [8–10].

### Causes of antimicrobial resistance

To better appreciate the causes of AMR, we need to understand the various sequential steps involved for a drug to get to a patient and the eventual use, which include; production, distribution, prescription, dispensing, and finally consumption of the drug by the patient or use in animal production [11]. Consequently, any imprudent practice along this flow may result in the emergence of resistance.

#### Drug dispensers and drug quality

The lack of appropriate regulations in the sales of antimicrobials is also a driving factor in the access and misuse of antimicrobials. In most developing countries, antimicrobials can be purchased without medical prescription [12] and are usually dispensed on the streets by untrained persons. These drug vendors will sell medications just to make a sale and accommodate patients’ ability to pay [13]. Even pharmacies operating without a license, appear to be more accessible to the public as they have shorter waiting time, do not charge consultation fees and above all are willing to negotiate treatment options to adjust to the financial ability of the patients [14]. Retail pharmacies in developing countries especially in Africa have emerged as the primary level of outpatient care rendering unauthorized services from consultation, diagnosis, prescription and dispensing of medication [14, 15].

It has also been shown that many antimicrobials dispensed in Africa are of questionable pharmacological quality [16, 17]. Adverse climatic conditions such as high ambient temperatures and humidity may affect the overall quality of the antimicrobials during storage [18, 19]. Poor storage also increases the risk of degradation of the drug. Degraded medicines contain less than stated dose, implying that patients consume less than optimal dose of the drug. There is also a problem of outright counterfeit, in which the drug may contain little or no active substance of the antimicrobial or the wrong substance [20]. The influx of counterfeit and sub-standard antimicrobials into the pharmaceutical markets in some regions is a major problem as these preparations of reduced potency also result in pathogens being exposed to sub-therapeutic concentrations of the drug [21]. A study from Cameroon revealed that, out of 284 antimalarial obtained from 132 vendors, 32% of chloroquine, 10% quinine, and 13% sulfadoxine/pyrimethamine were likely to be fake [16]. In addition, some of the quinine contained chloroquine while some chloroquine contained no active ingredient or an amount lower than the expected concentration.

#### Health professionals

Health care providers play an essential role in the treatment and prevention of diseases, but may jeopardize this if their practices are not evidence-based (Table 1). For example, the prescription practices of antimicrobials vary among physicians in most countries. In some cases, the antimicrobial prescriptions are inappropriate (i.e., wrong drug, wrong doses, or antimicrobial not necessary at all) [22]. Due to the high patient-doctor ratio in most developing countries, doctors are overwhelmed and there is often inadequate time for meaningful education and communication with the patient on drug adherence guidelines and consequences of poor or non-adherence to these guidelines. Treatment sometimes consists of administering broad-spectrum antibiotics without a definitive diagnosis and indication for antimicrobial treatment. In a Lebanese study, it was shown that in 52% of cases, the prescription dose was inappropriate while 63.7% of physicians prescribed antibiotics with wrong duration of treatment [23].

**Table 1:** Factors and stakeholders contributing to the problem of antimicrobial resistance

Factors	Contribution	Example
Poor drug quality	Sales of counterfeit, adulterated and poor quality antibiotics	These poor quality antibiotics can produce sub-inhibitory concentration in vivo, which increases the selection of resistant strains
Regulators	While most developed countries have developed AMR action plans, this is still lacking in many developing countries especially in Africa	Most countries lack the resources to enforce policies regarding the manufacture and distribution of sub-standard drugs
Prescribers	Excessive clinical use and misuse is partially responsible for increase rate of resistance	Variation in prescription practice among health care provider. Sometimes there is prescription of a wrong drug, wrong doses, or antimicrobial not necessary at all
Dispensers	Drug vendors usually have little or no knowledge of the required dosage regimen, indication, or contraindications	Medications are usually purchased in small aliquots from roadside stall and storage and distribution is usually done under inadequate conditions
Users (patients)	High rate of self- medication and lack of treatment compliance	Patients fail to adhere to dosage regimens and discontinue treatment when symptoms subside before pathogen is eliminated
Animal industry	The use of antimicrobial drugs in agriculture or industrial settings, exerts a selection pressure which can favor the survival of resistant strains (or genes) over susceptible ones, leading to a relative increase in resistant bacteria within microbial communities	Resistant bacteria in animals can be transferred to humans through the consumption of food or through direct contact with food-producing animals or through environmental spread

Due to lack of effective and reliable surveillance systems and poor dissemination of research information, health professionals in developing countries sometimes lack up to date information on the AMR pattern within their populations. In tertiary hospitals with advanced capacities, physicians rely mostly on the resistance or susceptibility pattern of the pathogen isolated from a patient. Health personnel in rural settings without the capacity to do AMR testing have difficulty to decide on the choice of antimicrobial in the absence of an antimicrobial sensitivity test. As a result, health professionals use more and more broad-spectrum antibiotics to treat infections caused by several bacteria species or those for which establishing the etiology is difficult or takes a long time. This practice contributes to the development of resistance [24] as the drug applies selective pressure, not only upon the etiological agent of the disease episode but also upon a large fraction of the patient’s microbiota [25]. Some health professionals issue prescriptions that are not evidence-based and rely on a syndromic approach to both infections in the community as well as hospitalized patients. That is treatment is based on easily recognized signs and symptoms (syndromes) as well as to microorganism most commonly responsible for each of these syndromes. This practice is on the rise due to the lack of legal consequences of wrongful prescription of antibiotics. Our preliminary findings in Cameroon revealed that a significant number of physicians are likely to prescribe antibiotics to treat diarrhea caused by rotavirus, due to the lack of capacity to establish the diagnosis (Unpublished). Due to fear of bad treatment outcomes of critical diseases, some physicians may resort to blind prescription of multiple and broad-spectrum antimicrobials. Meanwhile, because of financial incentives from drug suppliers, some physicians are prone to prescribing multiple antibiotics for the same condition [26].

#### Patients

As mentioned earlier, compliance is a major contributor to the development of AMR [27]. Patients miss doses, either by mistake or deliberate. Because patients are aware of the adverse impact of drinking alcohol while on antibiotics, some patients may skip doses when invited for a party in favor for the consumption of alcohol (unpublished data). These practices result in exposure of surviving microbes to sub-therapeutic concentrations of the drug and, consequently increases the chances of developing resistance [25]. Because of poverty, many sick individuals in developing countries of Africa often seek their first-line of treatment from traditional healers who provide them with herbal mixtures of unknown efficacy for the treatment of infections. Some combine antibiotics with their herbal mixtures simultaneously while others take antimicrobials and supplement them with herbal mixtures purportedly to improve efficacy [12]. These compounds of unknown potency may enhance pathogen fitness.

#### Non-human use of antimicrobials

Antimicrobials are used to prevent (prophylaxis in high risk animals) and treat diseases in animals, as well as used as growth promoters in animal breeding [28, 29]. Additionally, they are used as additives in plant agriculture (fruits, vegetables, and orchid, etc.), especially in the spraying of fruit trees for disease prophylaxis and the application of antibiotic-containing manure on farmland and in industrial processes [30]. The use of antimicrobial agents in animals and more importantly food-producing animals has important consequences for both human and animal health as it can lead to the development of resistant bacteria (Table 1). These resistant bacteria (with resistance genes) in animals can be transferred to humans through the consumption of food or through direct contact with food-producing animals or through environmental spread (e.g. human sewage and runoff water from agricultural sites). The use of antimicrobial drugs in health care, agriculture or industrial settings, exerts a selection pressure which can favor the survival of resistant strains (or genes) over susceptible ones, leading to a relative increase in resistant bacteria within microbial communities [31]. It is now known that increased AMR in bacteria affecting humans and animals is primarily influenced by an increase in the use of antimicrobials for a variety of purposes, including therapeutic and non-therapeutic purposes in animal production [32]. A strong association between agricultural use of antimicrobials and the development of resistance has been suggested [33], and it has been shown that the bulk of antimicrobials used worldwide are not consumed by humans but rather are given to animals for the purposes of food production [34].

Multidrug resistant bacteria have been detected in both meat and fresh produce [35] and in humans in contact with livestock in many African countries [36–38]. A study in Kenya revealed a high level of antimicrobial drug residues in meat meant for consumption [34]. These findings further demonstrate that food animals are a major reservoir of drug resistant bacteria and present a major risk for dissemination and transmission of antimicrobial resistant bacteria in Africa as well as many developing countries. A large proportion of the population in developing countries live in close proximity with animals, thus increasing the chances of transmission of resistant microorganisms from animals to humans through animal handling [39, 40]. In a recent study in rural Bangladesh animal healthcare providers responded they more often sought animal health care from pharmacies and village doctors, citing the latter two as less costly and more successful based on past performance [41]. In the absence of an effective animal healthcare system, villagers depend on informal healthcare providers for treatment of their animals. This may lead to suboptimal use of antimicrobials in such settings with unhygienic animal husbandry practices. In terms of aquaculture, heavy use of prophylactic antibiotics in aquaculture has been reported in developing countries and is a growing problem for human and animal health and for the environment [42].

Overall, limiting the routine use of in-feed antibiotics will improve human and animal health, by reducing the development and spread of antibiotic-resistant bacteria. Therefore, judicious use of antibiotics in healthcare and agricultural settings is essential to slow the emergence of resistance and extend the useful lifetime of effective antibiotics that are in existence today.

In other to minimize the risk of AMR due to non-human use of antimicrobials especially in animal production, information resources in developing countries need to be strengthened to support health professionals, patients, animal keepers and the public, so that the society can have a better understanding of the value and importance of antimicrobials, especially antibiotics. Taken together, the excessive use of antimicrobials in animals and agriculture and the associated public health consequences justifies the importance of limiting the inappropriate use of antimicrobials in both agriculture and the veterinary sector.

### Inadequate surveillance and limited laboratory antimicrobial susceptibility testing

The availability of routine antimicrobial susceptibility testing to provide information on resistance trends, including emerging resistance is very essential for routine clinical practice and for the development of effective policies against AMR. Antimicrobial susceptibility testing is not often performed in most rural laboratories due to lack of capacity. In the absence of patient-specific antimicrobial susceptibility testing, community-based antimicrobial surveillance data may be very useful to health care professionals in particular communities or regions to treat infections with specific susceptible antimicrobials. Such surveillance needs to be conducted regularly and continuously because resistance rates can vary in one region of a country over time.

### Antimicrobial resistance control strategies

The problem of AMR is aggravated by the fact that most world pharmaceutical companies consider research for new antimicrobials as being of “low profit” and some speculate that resistance will eventually develop for new antimicrobials anyway. Consequently, they prefer to invest in the development of drugs for chronic diseases (diabetes, hypertension) as well as those used to improve lifestyle (e.g., Cialis, Viagra, etc.) [43]. Therefore, the long-term solution should be focused on methods to prevent the emergence of resistance or the spread of resistant organisms from one person to another.

#### Hygiene and sanitation

Apart from the irrational use of antimicrobials, unique environmental conditions such as crowding and poor sanitation also contribute in the circulation and spread of resistant microorganisms. Transmission of resistant pathogens is facilitated by person-person contact, through contaminated water, food or by vectors. Improving basic hygiene and sanitation will reduce the spread of resistant organisms (Table 2) [44]. Improving infection prevention and control in hospitals will reduce the nosocomial spread of bacteria with acquired resistance such as *Staphylococcus aureus* amongst others.

**Table 2:** Strategies to contain and minimize the development of antimicrobial resistance

Control strategies	Contribution
Hygiene and sanitation	Improving basic hygiene and sanitation will reduce the spread of resistant organisms
Vaccination	Vaccination may reduce severity of disease, provide protection against shedding of pathogens and even raise the threshold load of pathogens required for infection
Alternative therapies	The reluctance of pharmaceutical companies to invest in research and development of novel antimicrobial agents necessitate the exploration of alternative therapies such as bacteriophage, probiotics and Quorum Sensing inhibitors
Education	Health care providers, dispensers and patients need to be educated on how the use and misuse of antimicrobial may contribute to the development of resistance
Infection prevention & control	Proper hospital infection control may prevent the spread of nosocomial pathogens and resistant microbes that may have easily been disseminated to the community if these measure were not in place

#### Vaccination

While antimicrobials are used for treatment, vaccines are a primary mode of prevention of infectious diseases. Prior vaccination may reduce severity of disease, provide protection against shedding of pathogens and even raise the threshold load of pathogens required for infection [45–47]. Also, indirect population protection of some vaccines as a result of herd immunity in unvaccinated individuals represents additional advantage of vaccination. In most regions of the world, a number of diseases such as smallpox, measles, mumps rubella, diphtheria, hepatitis A, pertussis, and polio have been prevented by vaccination [48].

However, even with the most effective vaccine, the need of antimicrobials or alternative treatment options will still in some cases be solicited. For example, in the case of genetic drift, escape mutants and serotype or strain replacement diseases, vaccination may fail to confer full protection from disease. For bacterial infections, the introduction of conjugate vaccines for example *Streptococcus pneumoniae,* has reduced the outbreak of respiratory infections in children in developing countries [49].

#### Alternative therapies

The increase in microbial resistance to traditional antimicrobials and the reluctance of pharmaceutical companies to invest in research and development of novel antimicrobial agents necessitate the exploration of alternative therapies. Future focus of medical therapeutics and research is to look beyond antibiotics [43], and search for alternatives which can regulate the microbial virulence as well as growth inhibition. There are currently a couple of other alternatives approaches at different levels of research and development.

1. The use of bacteriophage is emerging as an alternative treatment option for bacteria infections [50]. Many authors have suggested that bacteriophage therapy is a necessary alternative to conventional antibiotics [51, 52]. Bacteriophages are bacterial viruses with the capacity to invade bacterial cells and induce lysis of the bacteria (lytic cycle). In the present era of multidrug resistant bacteria and reluctance in the development of new antibiotics by pharmaceutical companies, the need to aggressively explore the possibility of phage therapy is unprecedented.
2. Quorum Sensing inhibitors represents an important antimicrobial target that may prevent, suppress, and/or treat infectious diseases. The mechanistic details (including auto-inducers) of Quorum Sensing are different between Gram-negative and Gram-positive bacteria. Gram-negative bacteria utilize *N*-acyl l-homoserine lactones (AHLs), which are homoserine lactone (HSL) rings with an additional fatty acid side chain while Gram positive bacteria uses oligopeptides [53–55]. While antibiotics kill or slow down the growth of bacteria, quorum sensing inhibitors or quorum quenchers simply attenuate bacterial virulence. A large body of work on Quorum Sensing has been carried out in deadly pathogens like *Pseudomonas aeruginosa, Staphylococcus aureus, Vibrio fischeri, Vibrio. harveyi, Escherichia coli* and *Vibrio. cholerae* etc. A number of these studies have succeeded in exploiting the bacterial Quorum Sensing system as potential target for treatment of bacterial infections. The inhibition of Quorum Sensing system is believed to be advantageous over conventional antibiotics, because only the communication mechanism between the bacteria is disrupted without killing the individual cells. Hence, this strategy should generate a lower selective pressure and reduce the rate at which AMR develops during the treatment [55].
3. Probiotics, otherwise referred to as fecal transplant therapy (FTT) is a treatment option that has been employed for decades, albeit with mixed results [56–58]. FTT is the act of using fecal material from pathogen-free healthy donors to repopulate the microbiota of a recipient. Probiotics are considered to be able to destroy pathogenic microorganisms by producing antimicrobial compounds such as bacteriocins and organic acids, improve gastrointestinal microbial environment by adherence to intestinal mucosa thereby preventing attachment of pathogens and competing with pathogens for nutrients, stimulate the intestinal immune responses and improve the digestion and absorption of nutrients. The commonly used probiotics include *Bacillus*, *Lactobacillus*, *Lactococcus*, *Streptococcus*, *Enterococcus*, *Pediococcus*, *Bifidobacterium*, *Bacteroides*, *Pseudomonas*, yeast, *Aspergillus*, and *Trichoderma*, etc.

This treatment option is an old practice, used many years ago, especially in China and had been successfully used to treat *Clostridium difficile* infections as well as other enteric diseases [56, 59, 60].

Taken together, the application of these alternate therapies is not limited to developing countries and most are still under development or at different stages of clinical trial and their routine availability will require governmental approval and subvention in developing countries.

### The role of stakeholders in the control of antimicrobial resistance

The control of AMR cannot be the sole responsibility of medical professionals and scientists. Although continuing education is essential to enable health care providers learn the need in rational prescription practices and the importance of evidence-based prescription [61], the general population and other stakeholders have a central role to play [62]. The government of every country should consider AMR as a public health priority issue. Policies and regulations should be put in place to enforce the prudent access and use of antimicrobials. The population needs to be educated on the threat of AMR and therefore media professionals need to get adequate training on how to convey medical and scientific information in lay language through multiple channels to inform the populations on practices that promote the emergence of AMR organisms [62]. The benefits of an education program for journalists has been successful for the fight against HIV/AIDS in many developing countries and a similar approach for AMR will likely result to an added benefit [63, 64]. A number of initiatives have been taken so far by regulatory agencies and governments in the combat of antimicrobial resistance but most of these initiatives have been done only in developed countries [65–68] and are lacking in resource limited nations [69, 70].

A few African countries have been identified as being involved in the Global Antibiotic Resistance Partnership (GARP), established in 2009 to create a platform for developing actionable policy proposals on antibiotic resistance in low-income and middle-income countries. Unfortunately, to date national GARP working groups are established only in a few African countries [70]. There is therefore need to generate solutions such as to build a strong African laboratory infrastructure to help combat AMR which is a global health threat [71]. A multi-disciplinary approach involving a wide range of partners is therefore needed to limit the risk of AMR and minimize its impact on human and animal health especially in developing countries.

### The role of government regulatory agencies

With the new and emergent issue regarding the development of AMR, government agencies globally have engaged in new action plans in order to combat the AMR issue [72]. However, actions toward this has been severely lacking in developing countries especially those in Africa where high quality regulatory agencies are lacking. In May 2015, the WHO endorsed a global action plan to tackle AMR [73]. In September 2014, the US government released a ‘National Strategy for Combating Antibiotic-Resistant Bacteria’ that included an executive order signed by the US president [68]. The action plan provided several goals and a roadmap to guide the Nation in rising to this challenge. In 2013, the United Kingdom released the ‘UK Five Year Antimicrobial Resistance Strategy (2013 to 2018)’ that sets out actions to address the key challenges to AMR [67]. Similarly, in 2015, the government of Canada also released the Federal Action Plan on antimicrobial resistance and use in Canada (Building on the federal framework for action) [66]. While many developed countries have developed such AMR action plans either nationally or at the regional level such as the European Union and Asia-Pacific region, this is still lacking in many developing countries especially in Africa [69]. A few African countries have been identified as being involved in the Global Antibiotic Resistance Partnership (GARP), established in 2009 to create a platform for developing actionable policy proposals on antibiotic resistance in low-income and middle-income countries. However, national GARP working groups are established only in India, Kenya, South Africa, Vietnam, Mozambique, Nepal, Tanzania and Uganda. Moreover, a close look at the national action plans showed that only one African country (Ethiopia), was identified by the WHO as having a national AMR action plan [74] .

What most developing countries especially those in Africa lack is not the legislation prohibiting the manufacture and distribution of sub-standard drugs but resources to enforce these policies and to impose penalties to defaulters. There is also lack of resources to identify counterfeit drugs or verify the quality of locally manufactured or imported drugs [75]. In addition, governments need to address the sale of antimicrobials without prescription and illegalize the dispensing of drugs by unauthorized and unqualified persons. There is also need for the population to be sensitized on the public health risks of AMR. A recent WHO survey revealed that while much activity is underway and many governments are committed to addressing the AMR problem, there are major gaps in actions needed across all 6 WHO regions to prevent the misuse of antibiotics and reduce spread of AMR [69]. Despite these challenges some progress is currently being made in setting up drug regulatory agencies in Africa. The effort is supported by the New Partnership for Africa’s Development (NEPAD) of the African Union and the WHO. Through the creation of the African Medicines Regulatory Harmonization (AMRH), it is envisioned that safe, good quality, efficacious and reasonably priced medicines will be available in Africa [76]. Recently, the African Society for Laboratory Medicine (ASLM) has engaged in activities to share research, debate, and generate solutions to build a strong African laboratory infrastructure to help combat common global health threats, like AMR as well as HIV/AIDS, and other medical conditions [71]. Therefore, a multi-disciplinary approach involving a wide range of partners is therefore needed to limit the risk of AMR and minimize its impact on human and animal health especially in developing countries.

## Conclusions

The irrational use of antimicrobials is certainly a complex and multifactorial problem in developing countries, and a proper understanding of the problem is necessary for effective control policies. Without effective antimicrobials, diverse medical procedures such as surgery, the care of premature infants, cancer chemotherapy, care of the critically ill, invasive diagnostic and treatment procedures, and transplantation medicine will be severely hampered with a corresponding increase in morbidity and mortality from secondary microbial infections. The challenge of global antimicrobial resistance is comparable to climate change and global warming. Therefore, as we seek to protect the climate for the future generation, it is our responsibility not to pass over to the next generation, microbial population that is resistant to antimicrobial agents that they are supposed to treat as the consequence is likely to be very dangerous.

## Abbreviations

AIDS: Acquired immunodeficiency syndrome
AMR: Antimicrobial resistance
FTT: Fecal transplant therapy
HIV: Human immunodeficiency virus
GARP: Global antibiotic resistance partnership
